# Efficacy and safety of EGFR‑TKIs plus Shenqi Fuzheng injection for non-small cell lung cancer patients with EGFR-sensitive mutations

**DOI:** 10.1007/s00432-022-04297-3

**Published:** 2022-08-26

**Authors:** Jia-li Wang, Chuan-sheng Chen, Zhi-rong Jia, Li-yun Miao, Jun Xie, Zhen-zhen Pan, Ya-lei Duan, Shuo Liu, Meng-jun Hou, Xuan-sheng Ding

**Affiliations:** 1 School of Basic Medicine and Clinical Pharmacy, China Pharmaceutical University, Nanjing, 211198 China; 2 Nanjing Medical University, Nanjing, 211166 China; 3 The Affiliated Drum Tower Hospital of Nanjing University Medical College, Nanjing, 210008 China; 4 The Affiliated Cancer Hospital of Anhui University of Science and Technology, Huainan, 232033 China

**Keywords:** Non-small cell lung cancer, EGFR-TKIs, Shenqi Fuzheng injection, Progression free survival

## Abstract

**Purpose:**

The aim of this retrospective study is to evaluate the impact on efficacy and safety between epidermal growth factor receptor tyrosine kinase inhibitors (EGFR-TKIs) alone and in combination with Shenqi Fuzheng injection (SFI) in patients with advanced NSCLC harboring epidermal growth factor receptor (EGFR) activating mutations.

**Methods:**

Retrospectively, information of 88 patients receiving EGFR-TKIs as first-line targeted treatment or in combination with SFI in the Affiliated Drum Tower Hospital of Nanjing University Medical College and the Affiliated Cancer Hospital of Anhui University of Science and Technology was collected. The primary endpoint was to assess progression-free survival (PFS) and safety of EGFR-TKIs alone or in combination with SFI.

**Results:**

Between January 2016 and December 2019, a total of 88 patients were enrolled in this research, including 50 cases in the EGFR-TKIs single agent therapy group and 38 cases in the SFI combined with EGFR-TKIs targeted-therapy group. The median PFS (mPFS) of monotherapy group was 10.50 months (95%CI 9.81–11.19), and 14.30 months (95%CI 10.22–18.38) in the combination therapy group. Compared to the single EGFR-TKIs administration, combinational regimen with SFI exhibited a lower incidence of rash and diarrhea in patients and was even better tolerated.

**Conclusions:**

SFI combined with the first-generation EGFR-TKIs are more efficient, can prominently prolong the PFS and attenuate the adverse reactions in patients with advanced NSCLC with EGFR-sensitive mutations.

## Introduction

According to recent estimates of international agency for research on cancer, lung cancer remains among the top diseases with high mortality worldwide. More than 80% of those cases were diagnosed as NSCLC with less than 20% survival rate, consisting of adenocarcinoma and squamous cell carcinoma as histological subtypes predominantly (Osmani et al. 2018; Sung et al. 2021). Clinically, the main treatment methods for lung cancer include surgical resection, radiotherapy, chemotherapy, molecular targeted therapy and immunotherapy (Ettinger et al. 2021; Nagasaka and Gadgeel 2018). Selecting corresponding targeted therapies according to different genes is the current treatment measure of NSCLC. EGFR, the most representative target, is mainly mutated in exon 18–21 like deletion of exon 19 and L858R mutation of exon 21 (Riely et al. 2006; Sakurada et al. 2006). In patients with EGFR-mutated NSCLC, EGFR-TKIs, the first-line agent, represented by gefitinib obtain obvious clinical responsiveness and greatly prolong the survival time of patients (Lynch et al. 2004; Prabhu and Devaraj 2017). Unfortunately, disease progression is developed gradually in the vast majority of patients 10–14 months after treating EGFR-TKIs is subject to resistance (Rocco et al. 2019; Wu et al. 2014). Subsequent in-depth studies by researchers found that the main reason for EGFR-TKIs resistance was the T790M mutation in exon 20 of EGFR gene (Sequist et al. 2011). AZD9291 is the third-generation irreversible EGFR-TKI that is selective for both EGFR-TKI sensitizing and T790M resistance mutation (Cross et al. 2014). But drug resistance is still inevitable on account of the C797S mutation (Thress et al. 2015). Given that the widespread drug resistance, it is particularly urgent to find a promising combination therapy to prolong PFS of EGFR-TKIs treatment presents.

The basic theory of traditional Chinese medicine (TCM) therapy pays attention to restoring balance by improving body’s resistance and preventive ability to cancer via ameliorating correlation among self-controlled systems (Lu et al. 2004). A large number of clinical studies have confirmed that the intervention of TCM is capable of elevating the clinical efficacy of patients, reducing the recurrence risk of patients with early tumors, enhancing immunity and decreasing adverse reactions (Fu et al. 2018; Wang et al. 2019; Xie et al. 2019). In TCM, lung cancer belongs to the categories of lung amassment and pulmonary retention. The application of TCM with the ability of “strengthening the body” and “eliminating evil” in lung cancer has gone from theory to practice (Li et al. 2021). TCM and its active ingredients with great anti-tumor activity augment the efficacy of EGFR-TKIs in NSCLC (Chen and Feng 2021; Sui et al. 2020; Tang et al. 2019; Yao et al. 2020). What deserve more attention that the combination of TCM and EGFR-TKIs as a treatment scheme for NSCLC provides a new perspective for improving patient prognosis.

Shenqi Fuzheng Injection (SFI), concocts from astragali and codonopsis (Dong et al. 2010), both of which exhibit immunomodulating and immunorestorative effects and anticancer activities (Fu et al. 2014; Gao et al. 2018). In real world clinical setting, there are accumulating studies about SFI accompanied by chemotherapy for treating various types of cancer like lung cancer (Dong et al. 2017), breast cancer (Liu et al. 2019) and colorectal cancer (Xu et al. 2017), are capable of improving clinical efficacy, immune function, performance status and reducing toxicity. Of note, SFI may play a pivotal role in the treatment of patients with advanced NSCLC based on lots of published trials on the significant anticancer synergy effects of SFI in combination with chemotherapy for treatment of NSCLC (Chen et al. 2019; Dedong et al. 2016). The efficacy and safety of SFI combined with chemotherapy have been obvious to all, but whether its combination with EGFR-TKIs can prolong the survival of NSCLC patients clinically and why the mechanism of delaying its drug resistance still need to be deeply explored. Previous study in our laboratory has proved that abnormal increase of cholesterol level in tumor cells is one of the main reasons for drug resistance of NSCLC to gefitinib (Chen et al. 2018). What’s more, our previous laboratory study has revealed that SFI combined with gefitinib can inhibit the proliferation and cloning of NSCLC resistant cells, and potentiate the binding of gefitinib to EGFR target depending on the suppression of MAPK/SREBP1 pathway (Pan et al. 2020). In view of these researches, exploring the clinical effect of SFI plus EGFR-TKIs regimen in patients with EGFR mutation and providing relevant data evidence for its use in the comprehensive treatment of NSCLC patients present an imperative task.

This paper presented a retrospective study for the sake of elucidating whether SFI in combination with first-generation EGFR-TKIs improved the PFS of patients with advanced NSCLC.

## Methods

### Research subjects

**Fig. 1:**
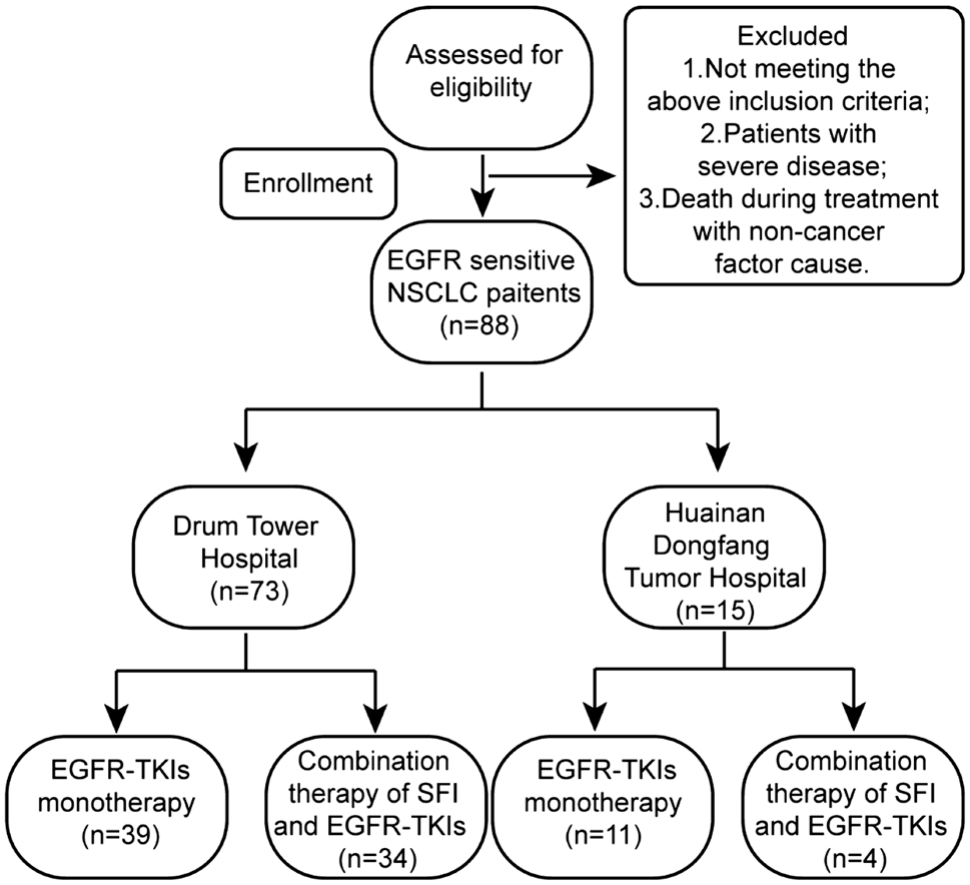
Study flow chart

From January 2016 to December 2019, 88 enrolled patients who were diagnosed with NSCLC in the Affiliated Drum Tower Hospital of Nanjing University Medical College, namely drum tower hospital and the Affiliated Cancer Hospital of Anhui University of Science and Technology, namely Huainan dongfang tumor hospital, affiliated with Anhui university of science and technology, were included in this retrospective study (Fig. 1). Eligible criteria were as follows: (1) patients aged older than or equal to 18 years confirmed by histopathological diagnosis of primary NSCLC, (2) all patients whose clinical stage is stage IIIB/IV according to the Eighth Edition of the TNM classification (Goldstraw et al. 2016), (3) all patients with an Eastern Cooperative Oncology Group performance status (ECOG PS) of 0–2, (4) none had received any anticancer therapy in the past, (5) none had second primary tumor disease, and (6) all patients with complete clinical data and detailed and comprehensive diagnosis and treatment records, including general conditions, treatment measures, use of TCM, etc. Exclusion criteria for patients as follows: (1) Those who do not meet the above inclusion criteria, (2) patients with severe cardiovascular disease, obvious coagulation dysfunction, active gastrointestinal bleeding and psychosis, and those with drug allergy, and (3) death during treatment with non-cancer factor cause. Clinical characteristics consisting of age, sex, pathological type, mutation type, stage, smoking history, and distal metastasis were recorded.

### Patients’ follow-up

The follow-up records of patients began from the time of diagnosis of NSCLC to December 31, 2020. All patients were followed up for PFS.

### Study design and treatment regimens

This study protocol was approved by the research ethics committee of Nanjing drum tower hospital and huainan dongfang tumor hospital. Approved by the State Food and Drug Administration of the People’s Republic of China in 1999, SFI was produced by livzon Pharmaceutics ltd, Zhuhai, China with the national medicine permission number (NMPN) of Z19990065. This study was based on the real world and was divided into groups according to different treatment schemes: (1) EGFR-TKIs alone group: in which patients received first-generation EGFR-TKIs gefitinib 250 mg QD, icotinib 125 mg TID and erlotinib 150 mg QD. Short-term delay, reduction or continuation of medication could be permitted according to individual conditions during treatment until disease progression; (2) SFI combined with EGFR-TKIs group: in which patients received EGFR-TKIs combined with SFI administered as an intravenous infusion 250 ml once daily for 21 days followed by first-line EGFR-TKIs, used 3 days before chemotherapy. SFI combined with EGFR-TKIs is utilized for patients returning to the hospital for in-hospital examination.

### Study evaluations

The primary end point was median PFS, referred to the time from the beginning of the EGFR-TKIs treatment to the progression of the disease. Chest computed tomography (CT) was performed in the following stages: before treatment, every two sessions during treatment or as necessary, until the disease progression. When the disease is suspected to progress, a systemic examination should be performed as early as possible to evaluate the current situation of tumor development. These test results were compared with each other to evaluate the growth rate or reduction rate of patients' tumors. Measured by response evaluation criteria in solid tumors (RECIST) v.1.1 (Eisenhauer et al. 2009), the efficacy evaluation of targeted therapy was categorized as complete response (CR), partial response (PR), stable disease (SD), and progression of disease (PD).

### Statistical analysis

Excel was used to establish a database, and SPSS 22.0 statistical software was used for statistical analysis. Significance in differences between groups was assessed by the chi-square test. Time-to-event variables were performed by the Kaplan–Meier method. The log-rank test was utilized for estimating survival rate. GraphPad Prism 9.0 software was used to draw the survival curve and a *P* value of < 0.05 indicated statistical differences.

## Results

### Patient disposition and characteristics

Eighty-eight patients with NSCLC were evaluated, whose main demographic and baseline characteristics are detailed in Table 1. Most of them (97.7%) suffered from lung adenocarcinoma at time of diagnosis. All patients harbored EGFR mutation with available assessment, among which 38.6% in deletion of exon 19 and 54.6% in L858R mutation of exon 21. According to the TNM staging standard of lung cancer formulated by International Association for the Study of Lung Cancer (IASLC), the clinical stages of patients were classified, including 2 cases of stage IIIB (2.3%) and 86 cases of stage IV (97.7%).

**Table 1:** Patient demographics and baseline characteristics (*n* = 88)

Characteristics	Number of cases (%)
Age (years)	
≥ 60	51 (58.0%)
< 60	37 (42.0%)
Sex	
Male	37 (42.0%)
Female	51 (58.0%)
Histological types	
Lung adenocarcinoma	86 (97.7%)
Squamous cell carcinoma of lung	2 (2.3%)
EGFR mutation status	
19del	34 (38.6%)
L858R	48 (54.6%)
Other	6 (6.8%)
Staging	
IIIB	2 (2.3%)
IV	86 (97.7%)
Smoking history	
Yes	21 (23.9%)
No	67 (76.1%)
Metastasis sites	
Lungs	44 (50.0%)
Liver	7 (8.0%)
Bone	48 (61.6%)
Brain	17 (19.3%)
Pleura	26 (29.5%)
Other	5 (5.7%)

### Efficacy of two therapy tumor response

**Fig. 2:**
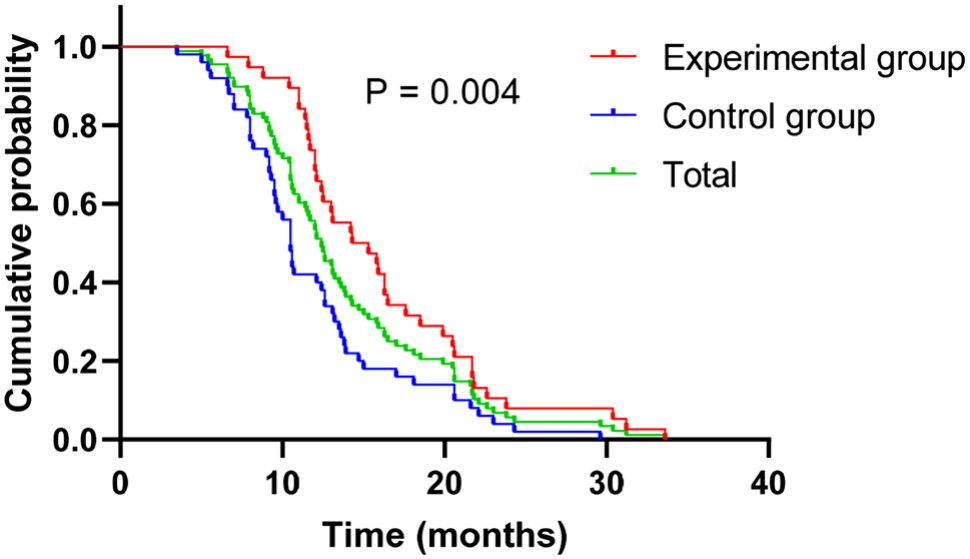
Kaplan–Meier curves of progression-free survival

At the time of this analysis, there were 38 cases (43.2%) in the trial group of SFI combined with the first-generation EGFR-TKIs and 50 cases (56.8%) in the single drug control group of the first-generation EGFR-TKIs. All patients reached PFS. The mPFS of the experimental group and the control group were 14.30 months (95% Cl 10.22–18.38) and 10.50 months (95% CI 9.81–11.19) (*P* = 0.004, Fig. 2), respectively. There was statistically significant difference, and of note, the efficacy of the combination group was prominently better than that of control group. Furthermore, all patients were included in mPFS for 12.40 months (95% CI 11.21–13.60, Fig. 2). The non-progression rate of the disease was 53.4% in 12 months and 22.7% in 18 months. Furthermore, data on responses to EGFR-TKIs monotherapy and co-administration of EGFR-TKIs with SFI targeted-therapy schedule according to RECIST are listed in Table 2.

**Table 2:** Responses to EGFR-TKIs monotherapy and co-administration of EGFR-TKIs with SFI targeted-therapy schedule according to RECIST (*n* = 88)

Best overall response	Number of cases (%)
Complete response	0 (0.0%)
Partial response	48 (54.5%)
Stable disease	40 (45.5%)
Progressive disease	0 (0.0%)

### Efficacy of each hospital

**Fig. 3:**
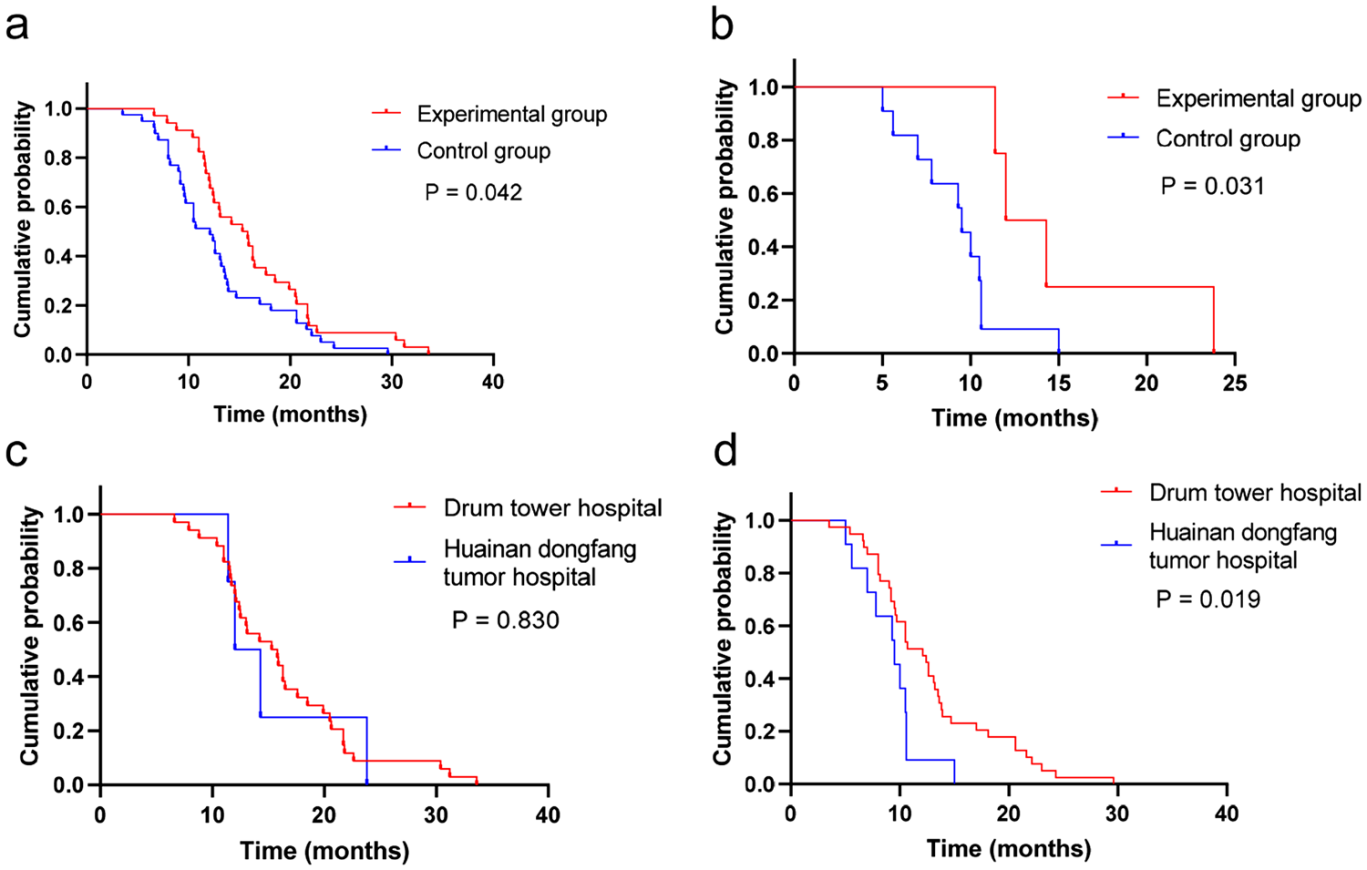
Kaplan–Meier curves of progression-free survival of each hospital. **a** Progression free survival curve of combination group and single agent targeted-therapy group in drum tower hospital. **b** Progression free survival curve of combination group and single agent targeted-therapy group in huainan dongfang tumor hospital. **c** progression-free survival curve of the combination group of drum tower hospital and huainan dongfang tumor hospital. **d** progression-free survival curve of single drug targeted treatment group of drum tower hospital and huainan dongfang tumor hospital

To decrease the divergence of PFS caused by the difference of medical level between different hospitals, the PFS data of each hospital were analyzed separately. There were 73 patients in drum tower hospital and all of them reached PFS, which were 46.5% in the experimental group and were 53.4% in the control group. PFS was significantly longer in the experimental group (15.3 (95% Cl 11.30–19.30) vs. 12.10 months (95% Cl 8.88–14.31) in the control group; *P* = 0.042, Fig. 3a). A total of 15 patients were included in huainan dongfang tumor hospital, including 4 cases in the experimental group (26.7%) and 11 cases in the control group (73.3%). All 15 patients reached PFS. The mPFS of the test group and control group were 12.00 months (95% Cl 9.16–14.84), and 9.50 months (95% Cl 7.13–11.87) (*P* = 0.031, Fig. 3b), respectively. In addition, the difference of mPFS between the same medication groups of drum tower hospital and huainan dongfang tumor hospital was compared. The results showed that the mPFS of drum tower hospital and huainan dongfang tumor hospital were 15.30 months (95% Cl 11.30–19.30) and 12.00 months (95% Cl 9.16–14.84) (*P* = 0.830, Fig. 3c). Under the single targeted treatment scheme, the mPFS of drum tower hospital and huainan dongfang tumor hospital were 12.10 months (95% Cl 8.88–14.31) and 9.50 months (95% Cl 7.13–11.87) (*P* = 0.019, Fig. 3d), respectively.

### Safety outcomes

Safety is of great importance as efficacy in assessing the role of an innovative combination due to most patients with advanced NSCLC ultimately die of their cancer, such as SFI plus EGFR-TKIs studied in this current research. The frequent adverse effects occurred during the administration are summarized in Table 3. Hypokalemia occurred in 4 patients (10.5%) in the experimental group and 2 patients in the control group (3.8%). 20 patients (52.6%) experienced abnormal liver function in the experimental group and 26 patients (52.0%) in the control group. Pneumonia induced in 4 patients (10.5%) in the experimental group and 2 patients (4.0%) in the control group. However, in the combination, there was a decrease in diarrhea (4 patients in the experimental group (10.5%) versus 12 (24.0%) in the control group), together with a decrease in rash (9 patients (23.7%) in the experimental group versus 15 patients (30.0%) in the control group).

**Table 3:** Safety outcomes

	Group	
	Experimental group (*n* = 38)	Control group (*n* = 50)
Hypokalemia		
Yes	4 (10.5%)	2 (4.0%)
No	34 (89.5%)	48 (96.0%)
Nausea		
Yes	4 (10.5%)	5 (10.0%)
No	34 (89.5%)	45 (90.0%)
Fatigue		
Yes	2 (5.3%)	3 (6.0%)
No	36 (94.7%)	47 (94.0%)
Diarrhea		
Yes	4 (10.5%)	12 (24.0%)
No	34 (89.5%)	38 (76.0%)
Abnormal liver function		
Yes	20 (52.6%)	26 (52.0%)
No	18 (47.4%)	24 (48.0%)
Rash		
Yes	9 (23.7%)	15 (30.0%)
No	29 (76.3%)	35 (60.0%)
Interstitial pneumonia		
Yes	4 (10.5%)	2 (4.0%)
No	34 (89.5%)	48 (96.0%)
Myelosuppression		
Yes	0 (0)	2 (4.0%)
No	38 (100%)	48 (96.0%)

## Discussion

Clinically, EGFR-TKIs targeted therapies (gefitinib, erlotinib, icotinib) result in very favorable outcomes with PFS of 10–13 months for patients with advanced NSCLC harboring somatic EGFR mutations (Exon 19-del, Exon 21 L858R) (Sequist et al. 2008). Whereas, with continued administration duration, most patients emerge acquired resistance to targeted drugs, approximately 60% of which were acquired EGFR 20 exon T790M mutation (Herbst et al. 2018). Although EGFR-TKIs are currently developed to fourth generation, the inevitable problem of acquired drug resistance has been plaguing clinical researchers. In recent years, owing to the multi-target, multi-ingredient, multi-level and mild characteristics of proprietary TCM, growing researchers have turned to the study of proprietary medicine and antineoplastic agents combination regimen (Tang et al. 2021; Zhang et al. 2020). Based on therapeutic advantages verified repeatedly in clinical practice, TCM plays a vital role in the malignant tumor treatment system that cannot be ignored in Chinese medical structure for tumors, especially in the treatment of chronic disease. TCM advocates that the treatment of cancer must pay attention to the deficiency of healthy energy, emphasizes the benefit of nourishing positive accumulation, which in turn improve the patient's deficiency state and then strengthen the body's ability to resist diseases. SFI is widely used in clinical applications, which can effectively improve the hematopoietic function, immune function of patients and the survival quality of patients, as well as increase the effectiveness and reduce the toxicity of radiotherapy (Meng et al. 2022). The combination of chemotherapy and SFI exhibits good benefit in patients with recurrent metastatic or advanced (stage IIIB/IV) NSCLC, as well as remarkably enhances Qi insufficiency constitution (Liu et al. 2022). Our former work showed capability of SFI to ameliorate therapeutic efficiency of gefitinib (Pan et al. 2020). Therefore, it is fulfilling to investigate the association between SFI and EGFR-TKIs resistance in clinic, which has been rarely revealed.

In our real-life series, SFI was clinically effective in prolonging duration of first-generation EGFR-TKIs. We observed a mPFS of 12.40 months (95% Cl 11.21–13.60) in whole group, with 14.30 months (95% Cl 10.22–18.38) in the combination group and 10.50 months (95% Cl 9.81–11.19) in the group only administered EGFR-TKIs alone (*P* = 0.004), conferred a statistically significant. In addition, the mPFS of treatment with SFI combined with first-generation EGFR-TKIs was significantly better than the mPFS of first-generation EGFR-TKIs (gefitinib: 9.6 months; erlotinib: 13.3 months; icotinib: 11.2 months) in series of phase III clinical trials results (Kelly et al. 2019; Saito et al. 2019; Shi et al. 2017), suggesting that this combination regimen are capable of effectively controlling tumor progression in advanced NSCLC patients harboring EGFR-sensitive mutations, producing significantly longer PFS, and slowing down the onset of drug resistance. In addition, in this study, to circumvent the disparity of medical level between different hospitals, the data of patients included in drum tower hospital and huainan dongfang tumor hospital were each analyzed separately. The mPFS was 15.30 months (95% Cl 11.30–19.30) in the combination group and 12.10 months (95% Cl 9.88–14.32) (*P* = 0.042) in control group at drum tower hospital. The mPFS was 12.00 months (95% Cl 9.16–14.84) in the combination group and the median PFS was 9.50 months (95% Cl: 7.13–11.87, *P* = 0.031) in the control group at Huainan Dongfang tumor hospital. The results were consistent with the outcome of the above study results, all manifesting the delaying resistance ability of SFI in NSCLC patients. In the meantime, whether the difference in treatment level between different hospitals under the same treatment had an impact on PFS of patients was investigated, and the results showed that the difference in PFS between two hospitals for the administration regimen of SFI combined with EGFR-TKIs was not significant. Of note, there was a significant difference in PFS between monotherapy different from combination therapy, suggesting that the difference in treatment level between different hospitals may affect patients' PFS, which probably result from the small number of patients enrolled in huainan dongfang tumor hospital. Next, we should dig deeper into focus on how SFI functions in clinical NSCLC patients during hospitalization and its appropriate medicine dosage and administration time to overcome the current clinical problems of injectable intakes of SFI. Meanwhile, further evidence is also required to explore the sensitive factors influencing the prognosis of treatment resistance of the first-generation EGFR-TKIs and which factors SFI affects to improve the prognosis of patients.

SFI is mainly used for the adjuvant treatment of lung cancer and gastric cancer as a large infusion solution widely used in clinical application, can improve fatigue-like behavior in cancer-related fatigue mouse models by inhibiting the production of pro-inflammatory cytokines produced by peripheral immune cells, inhibit the dysfunction of failing T cells, and improve the body’s anti-tumor immunity by targeting targets such as PD-L1, TIM3 and FOXP3 (Zhu et al. 2019). It is important to notice that SFI prominently reduces the incidence of rash and diarrhea in patients. However, for liver dysfunction, interstitial pneumonia, nausea, fatigue and other symptoms caused by EGFR-TKIs, there was no significant difference between combination regimen and EGFR-TKIs alone. Since this retrospective study carries some weaknesses such as the number of enrolled patients was small and the clinical medical records did not indicate the severity of adverse events, we must interpret our results carefully as they possibly be suffered from prejudice. Therefore, a prospective clinical study of large sample sizes is still necessitated to investigate whether SFI are able to reduce the incidence and severity of adverse reactions in the first-generation of EGFR-TKIs. Limitations remain and clinical benefits require further high-quality trials to verify. Whereas, the definite efficacy of SFI on delaying gefitinib resistance is not yet clear.

In conclusion, for NSCLC patients with EGFR-sensitive mutations (Exon19 Del, L858R), patients treated with co-administration of EGFR-TKIs with SFI achieve more prolonged clinical benefit and longer PFS than treated with the first-generation of EGFR-TKIs alone, and SFI clinically effectively retards the occurrence of first-generation EGFR-TKIs resistance. Synergistic treatment with SFI and EGFR-TKIs as an underlying means of augmenting the profit of EGFR-TKIs therapy, deeply warrants clinical investigation.

## Data Availability

Data sharing is not applicable to this article as no datasets were generated or analyzed during the current study.

## Abbreviations

ADR: Adverse reactions
CR: Complete response
CT: Computed tomography
DMEM: Dulbecco’s modified eagle medium
ECOG PS: Eastern cooperative oncology group performance status
EGFR: Epidermal growth factor receptor
EGFR-TKIs: EGFR tyrosine kinase inhibitors
IASLC: International association for the study of lung cancer
MTT: Methyl thiazolyl tetrazolium
mPFS: Median PFS
NSCLC: Non-small cell lung carcinoma
PD: Progression of disease
PFS: Progression free survival
PR: Partial response
RECIST: Response evaluation criteria in solid tumors
RI: Resistant index
SD: Stable disease
SFI: Shenqi Fuzheng injection
TCHM: Traditional Chinese herbal medicine
TCM: Traditional Chinese medicine
